# Distribution of Insecticide Resistance Genetic Markers in the West Nile Virus Vector *Culex pipiens* from South-Eastern Romania

**DOI:** 10.3390/insects13111062

**Published:** 2022-11-17

**Authors:** Ioana Georgeta Stancu, Florian Liviu Prioteasa, Georgiana Victorița Tiron, Ani Ioana Cotar, Elena Fălcuță, Daniela Porea, Sorin Dinu, Cornelia Svetlana Ceianu, Ortansa Csutak

**Affiliations:** 1 Department of Genetics, Faculty of Biology, University of Bucharest, 1–3 Aleea Portocalelor, 060101 Bucharest, Romania; 2 Vector-Borne Infections Laboratory, Cantacuzino National Military Medical Institute for Research and Development, 103 Splaiul Independenței, 050096 Bucharest, Romania; 3 Medical Entomology Laboratory, Cantacuzino National Military Medical Institute for Research and Development, 103 Splaiul Independenței, 050096 Bucharest, Romania; 4 Department of Microbiology, Faculty of Biology, University of Bucharest, 1–3 Aleea Portocalelor, 060101 Bucharest, Romania; 5 Danube Delta National Institute for Research and Development, 165 Babadag, 820112 Tulcea, Romania; 6 Molecular Epidemiology for Communicable Diseases Laboratory, Cantacuzino National Military Medical Institute for Research and Development, 103 Splaiul Independenței, 050096 Bucharest, Romania

**Keywords:** *Culex pipiens*, *pipiens* biotype, *molestus* biotype, insecticide resistance, pyrethroids

## Abstract

**Simple Summary:**

*Culex pipiens* mosquitoes are the vectors of West Nile virus in south-eastern Romania, a region where significant outbreaks of infection with this virus have occurred since 1996. The mosquito control strategy in Romania consists of using chemical insecticides, usually after the onset of the first human infection cases, but with limited impact. The level of insecticide resistance in the mosquito populations in the investigated area has not been assessed previously. We screened mosquitoes for mutations associated with resistance to the most used categories of insecticides: organophosphates, carbamates, and pyrethroids. Prior to this, the biotype of each mosquito specimen was determined. Low-frequency resistance mutations to organophosphates and carbamates were detected in *Culex pipiens molestus* mosquitoes collected in urban areas. High frequencies of pyrethroid resistance mutations were found in the *pipiens* and *molestus* biotypes populations and also in the hybrids collected in urban and in intensive agriculture areas. We recommend limiting the use of pyrethroids, when possible, and continuous monitoring of insecticide resistance mutations in mosquito populations in south-eastern Romania.

**Abstract:**

*Culex pipiens pipiens* and *Culex pipiens molestus* mosquitoes are the vectors of West Nile virus in south-eastern Romania, an area of intense circulation and human transmission of this virus. The level of insecticide resistance for the mosquito populations in the region has not been previously assessed. *Culex pipiens* mosquitoes collected between 2018 and 2019 in south-eastern Romania from different habitats were subjected to biotype identification by real-time PCR. Substitutions causing resistance to organophosphates and carbamates (F290V and G119S in acetylcholinesterase 1) and to pyrethroids (L1014F in voltage gated Na^+^ channel) were screened by PCR or sequencing. Substitutions F290V and G119S were detected at very low frequencies and only in heterozygous state in *Culex pipiens molestus* biotype specimens collected in urban areas. The *molestus* biotype population analysed was entirely homozygous for L1014F, and high frequencies of this substitution were also found for *pipiens* biotype and hybrid mosquitoes collected in urban and in intensive agriculture areas. Reducing the selective pressure by limiting the use of pyrethroid insecticides only for regions where it is absolutely necessary and monitoring L1014F mutation should be taken into consideration when implementing vector control strategies.

## 1. Introduction

West Nile virus (WNV) is endemic in south-eastern Romania, where large outbreaks of human infection have been reported since 1996 [1,2,3]. The virus is transmitted in Romania by *Culex pipiens* mosquito vectors and is mainly amplified by passerine hosts [1,4,5]. The two biotypes of this species—*pipiens* and *molestus*—and their hybrids have a sympatric distribution in the area but play different roles in the transmission cycles of WNV, as seen from habitat preference and host-feeding pattern studies; *pipiens* biotype mosquitoes act as enzootic/epizootic vectors, whereas *molestus* biotype and hybrids play a significant role as bridge vectors [5].

Currently, the mosquito vector control strategy in Romania consists of using chemical insecticides, usually after the onset of the first WNV human cases. Environmental-friendly bacterial larvicides are used only in the urban natural wetland reserve. The impact of this control strategy is limited since the outbreaks follow their natural dynamics in summer months until the end of September [1,2,3,6].

A recent global survey collecting data from 87 countries and analysing the use of vector control insecticides in spraying operations between 2010 and 2019 showed that organochlorines (OC), organophosphates (OP), carbamates (CX), and pyrethroids (PYR) are the most used classes of insecticides worldwide [7].

As reviewed elsewhere [8], multiple insecticide resistance mechanisms have been identified in mosquito species. Increased metabolic detoxification of insecticides and decreased sensitivity of the target proteins are the most significant resistance mechanisms and have been intensely studied. Decreased sensitivity of target proteins or target-site modification/insensitivity is acquired by point mutations in genes encoding insecticide target proteins, resulting in reduced binding of the insecticide.

G119S and F290V substitutions occurring in the active site of acetylcholinesterase 1 (AChE1)—a key enzyme in synaptic transmission encoded by *ace-1* gene—cause reduced sensitivity or resistance to OP and CX insecticides in mosquito species [9,10,11,12,13].

Substitution L1014F in the voltage gated Na^+^ channel (VGSC) determines knockdown resistance (*kdr*) to PYR in *Culex pipiens* mosquitoes [14]. *Kdr* is a cross-resistance to the organochlorine DDT (dichloro-diphenyl-trichloroethane) and to PYR and was described for the first time in a house fly strain [15]. Later studies confirmed the presence of L1014F substitution in *Culex* mosquitoes in different countries [16,17,18,19].

The aim of this study was to investigate the presence of insecticide resistance mutations in *Culex pipiens* vector populations from a WNV endemic area in south-eastern Romania so as to provide data to be used in operational mosquito control strategies in the area.

## 2. Materials and Methods

### 2.1. Study Sites and Sample Collection

**Figure 1:**
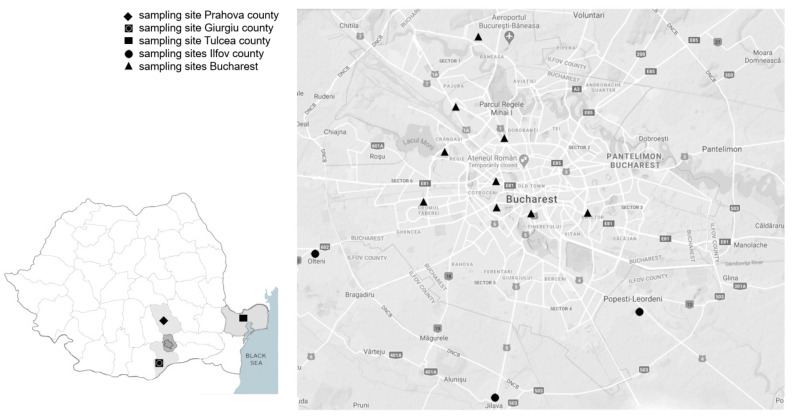
Map of mosquito sampling sites, south-eastern Romania, 2018–2019.

Mosquito collections were carried out between 2018 and 2019 from fifteen sites located in south-eastern Romania, mainly in the framework of WNV surveillance. Adult mosquitoes were collected in Bucharest city (nine sites), Ilfov county (three sites), and Giurgiu county (one site) using Centers for Disease Control and Prevention (CDC) gravid traps (John W. Hock Company, Gainesville, FL, USA). Overwintering mosquitoes were collected in Tulcea county (one site) using hand aspirators. Larvae were collected in one site in Prahova county (Table 1, Figure 1). Most of the collections were performed in Bucharest city and the adjacent Ilfov county. This area is located in the Danube plain and displays a wide variety of mosquito habitats ranging from administrative and residential areas to parks, bodies of water, a wetland nature park, forests, and industrial and agricultural areas. Adult mosquitoes were morphologically identified using a key described by Becker [20]. *Culex pipiens* s.l. females and larvae were further taken into study.

### 2.2. Biotype Identification

Individual adult mosquitoes/larva were homogenized in 0.2 mL PBS solution and used for DNA extraction with a commercial kit (ReliaPrep™ Blood gDNA Miniprep System, Promega, Madison, WI, USA) following the manufacturer′s protocol. The DNA was stored at −20 °C until further analysis. The biotype was assessed by a multiplex real-time PCR assay based on CQ11 microsatellite [21]. PCRs were carried out in a final volume of 25 µL using 2.5 µL of DNA and SensiMix™ II Probe Kit (Meridian Bioscience, Cincinnati, OH, USA). Primers and probes were used at a concentration of 0.4 µM and of 0.1 µM, respectively. The amplification (95 °C for 10 min; 45 cycles of 95 °C for 10 s, 60 °C for 1 min) was run on Mx3005P Real-Time PCR System (Agilent, Santa Clara, CA, USA).

### 2.3. Detection of AChE1 F290V Substitution

F290V substitution was detected using an allele-specific PCR protocol [12]. PCRs were carried out in a final volume of 25 µL using 5 µL of DNA and GoTaq^®^ Green Master Mix (Promega, Madison, WI, USA). Primers CxEx5dir, CxKrev2, and Valdir were used at a concentration of 0.8 µM, whereas Valrev primer was used at a concentration of 0.4 µM. The amplification (95 °C for 2 min; 30 cycles of 95 °C for 30 s, 51 °C for 30 s, 72 °C for 1 min) was run on DNA Engine^®^ Thermal Cycler (Bio-Rad, Hercules, CA, USA). The resulting PCR products were subjected to electrophoresis in 1.5% agarose gel and visualised under UV light after staining with ethidium bromide.

### 2.4. Detection of AChE1 G119S Substitution

A 511 bp DNA fragment containing codon 119 of *ace-1* was amplified using primers described before [10] and sequenced on a SeqStudio™ Genetic Analyzer System using BigDye™ Terminator v3.1 Cycle Sequencing Kit (Applied Biosystems, Waltham, MA, USA).

### 2.5. Detection of L1014F Kdr Mutation

For the detection of L1014F *kdr* mutation, we used the method described by Martinez-Torres et al. [14]. PCRs were carried out in a final volume of 25 µL using 5 µL of DNA and GoTaq^®^ Green Master Mix (Promega, Madison, WI, USA). Primers Cgd1, Cgd3, and Cgd4 were used at a concentration of 0.4 µM, whereas primer Cgd2 was used at a concentration of 0.8 µM. The amplification (95 °C for 2 min; 30 cycles of 95 °C for 30 s, 48 °C for 30 s, 72 °C for 1 min) was run on DNA Engine^®^ Thermal Cycler (Bio-Rad, Hercules, CA, USA). The resulting PCR products were analysed in 1.5% agarose gel and visualized under UV light after staining with ethidium bromide. To check the specificity of the method, 10 randomly selected samples genotyped as homozygous were also PCR amplified using primers Cgd1 and Cgd2 [14] and sequenced as described above.

## 3. Results

### 3.1. Biotype Identification

Approximately 11,000 mosquitoes were collected between 2018 and 2019 in the fifteen analysed sites, most of them being pooled and analysed for the presence of WNV. A convenience sample of 271 mosquito specimens (261 adults and ten larvae morphologically identified as *Culex pipiens* s.l.) was subjected to biotype identification. From the sites where a low number of mosquitoes were collected, all the specimens were included in the study (i.e., Giurgiu, Tulcea, and Prahova sites). In the limits of the budget, the other samples were randomly chosen from Bucharest and Ilfov county. Multiplex real-time PCR assay results showed that 166 of the tested specimens belong to *pipiens* biotype, 90 to *molestus* biotype, and 15 are *pipiens* × *molestus* hybrids.

### 3.2. Detection of AChE1 F290V Substitution

**Figure 2:**
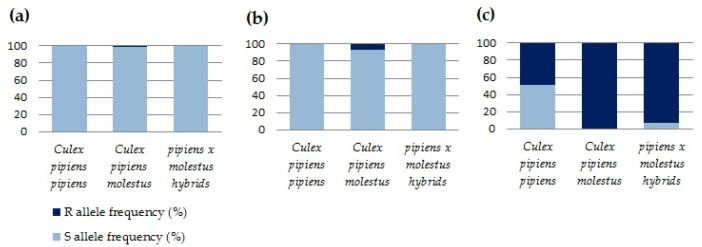
Allele frequencies for insecticide resistance genetic markers analysed in this study. (**a**): S—allele encoding for phenylalanine (F) at codon 290 of acetylcholinesterase 1 (AChE1); R—allele encoding for valine (V) at codon 290 of AChE1; (**b**): S—allele encoding for glycine (G) at codon 119 of AChE1; R—allele encoding for serine (S) at codon 119 of AChE1; (**c**): S—allele encoding for leucine (L) at codon 1014 of voltage gated Na^+^ channel (VGSC); R—allele encoding for phenylalanine (F) at codon 1014 of VGSC.

F290V substitution conferring resistance to OP and CX was assessed for the entire convenience sample. This mutation was found only in two *Culex pipiens molestus* specimens (1.1% allele frequency) collected in Bucharest city and only in heterozygous state (Table 2, Figure 2a).

### 3.3. Detection of AChE1 G119S Substitution

As we detected G119S substitution by sequencing, its presence was examined in only 43 mosquitoes from the convenience sample, due to financial limitations. The allele conferring resistance to OP and CX was found in other two heterozygous *Culex pipiens molestus* specimens collected in Bucharest, at a frequency of 6.7% (Table 3, Figure 2b). Since the obtaining sequences also spanned codon 290, the results for the allele-specific PCR used for F290V detection were confirmed for these 43 samples.

### 3.4. Detection of L1014F Kdr Mutation

The presence of L1014F *kdr* mutation was assessed for the entire convenience sample. The *kdr* allele was found in all the analysed populations, except in the samples collected in Prahova county, with frequencies ranging from 49.4% in the *pipiens* biotype to 100% in the *molestus* biotype (Table 4, Figure 2c). The frequency of the *kdr* homozygous individuals was high as 30.7% for *pipiens* biotype, 86.7% for *pipiens* × *molestus* hybrids, and 100% for the *molestus* biotype. The sequencing results for the ten samples genotyped as homozygous confirmed the results of the allele-specific PCR.

## 4. Discussion

In our study, G119S and F290V AChE1 substitutions responsible for OP and CX resistance were found only in the heterozygous state, each in two different *Culex pipiens molestus* specimens collected in Bucharest city. The low frequency of the two mutations in the analysed mosquito population in south-eastern Romania are consistent with the findings of previous studies conducted on mosquito populations in Greece [17,18] but differs from a study in Algeria [22], where the selective pressure of OP and CX was relatively significant. The similarities between south-eastern Romania and Greece could be explained by the fact that OP and CX insecticides have been banned in Europe, and their use has been dramatically interrupted. In the absence of the selective pressure exerted by insecticides, few mutations are maintained in the population, given the fact they might elicit a fitness cost. In a study conducted in Lebanon [23], a significant reduction was observed in the frequency of G119S substitution after the shift from OP insecticides towards the use of PYR. Furthermore, F290V substitution was not detected in the mosquito population in Lebanon. There have been numerous studies that demonstrated the fitness costs of *ace-1* resistance, such as an increased larval predation risk [24] or a longer larval development time [25].In a study conducted in urban areas in Morocco where temephos (OP) was used to control mosquito larval population, G119S substitution was observed at a relatively low frequency, but was significantly higher than the one found in the present study [26]. However, the size of the sample analysed in our study could have led to this difference. In the same country, another recent study [27] showed that both G119S and F290V substitutions were present at low frequencies, ranging from 0.08 (Mohammedia region) to 0.24 (Larache) and less than 0.01 (Agadir) to 0.19 (Larache), respectively. 

In Europe, the only adulticide accepted for the prevention of vector-borne diseases is PYR. Moreover, the insecticides used in vector control programs are the same ones used in household and for agriculture [17]. As a result, there is an increase in genetic resistance to insecticides in the vector population due to the selective pressure represented by continuous usage of the same chemical treatments [28]. Therefore, the monitoring of resistance to this class of biocides is critical for the success of the operations. The main genetic marker for PYR resistance is *kdr* resistance. The frequency of the *kdr* allele in *Culex pipiens pipiens* differs significantly between the four geographic regions investigated in our study. The highest frequency of mutant allele was recorded for mosquito population in Bucharest (66.8%), while, in a hilly village from Prahova county, we found only the wild homozygous genotype, which shows that the population was not subjected to selective pressure from insecticides. Indeed, the village is placed in a natural environment in which animal husbandry and bee-keeping are the main occupations of the inhabitants. Meanwhile, in the urban and peri-urban environment, there is an extensive use of PYR for personal protection indoors and outdoors for national pest control operations. The prolonged exposure to PYR during the control measures may have induced a selective pressure for the *kdr* allele, which became fixed after several generations. In contrast, in the village from Giurgiu rural area, the frequency of *kdr* allele was lower (38.9%) than the frequency encountered in the Bucharest metropolitan area (66.8%) but significantly higher than for Tulcea city (11.3%). Even though the selective pressure in the rural area determined by insecticides should not occur, Giurgiu is an area of intensive agriculture where crops are treated with the same types of insecticides as those used for vector control [29]. Only overwintering mosquitoes collected at the end of winter 2018 were analysed from Tulcea county. The *kdr* allele was found in this population at a reduced frequency (11.3%), with no mutant homozygous specimens detected. Given the fact that this mutation is located in a conserved domain of the protein, it might generate a fitness cost, and in the absence of selective pressure from insecticides, the frequency of the mutation can be diminished [30]. During the winter season, the hibernating state of the mosquitoes could be affected by the presence of the *kdr* mutations. A seasonal variation in the frequencies of *kdr* resistance mutations was also observed, which may be due to a fitness advantage of the susceptible genotype [31]. However, the reduced sample size in our study could have caused a distorted observation.

The *kdr* allele frequency was 100% in the *Culex pipiens molestus* population analysed, which might be an indicator of its homogeneity. These results differ significantly from a study conducted in Morocco, where the *kdr* allele was not found in *molestus* individuals [32]. Another study [19] conducted in the same area confirmed that the *kdr* allele is widespread in the *pipiens* biotype and hybrids, but no *kdr* homozygous were found among *molestus* biotype individuals. Another study from North America found that 52% of the *molestus* individuals—assignment based on habitat, morphology, and physiological traits—in a feral population of a mosquito displaying resistance to PYR were homozygous for the resistant allele [16]. Our finding that the *molestus* biotype population in the studied area is 100% homozygous for *kdr* is of high importance and shows that the mutated allele becomes fixed. The study conducted by Chen et al. in 2010 [33] demonstrated that the L1014F substitution becomes fixed after twelve generations in the *Culex pipiens pallens* under continuous exposure to deltamethrin. Our results indicate that in the Bucharest metropolitan area and in the Giurgiu rural area—where the *molestus* and hybrid populations were found—the measures for adult mosquito control should include the use of alternative active ingredients that will not contribute to knockdown resistance selection. Moreover, alternative control measures targeting the limitation of pre-adult populations, such as habitat reduction and larval control, should be used. Nonetheless, in these areas, routine monitoring for the presence of the *kdr* allele should also be included in the vector control program.

A high *kdr* allele frequency (93.3%) was also found in the *pipiens* × *molestus* hybrids. Although the number of the hybrids analysed in this study is low, the data we obtained are worrying given their feeding behaviour similar is to that of *molestus*, which acts a bridge vector in the WNV’s transmission cycle [5].

## 5. Conclusions

To the best of our knowledge, we describe for the first time the genetic markers associated with resistance to the major classes of insecticides in mosquito population in south-eastern Romania, an area endemic for WNV. The molecular investigation performed in this study showed a high frequency of the L1014F *kdr* mutation, particularly in the *molestus* biotype and hybrids, and a low frequency of the mutations associated with OP and CX resistance. Based on the results of this study, we strongly recommend the use of PYR only in regions where it is absolutely necessary, thereby reducing the selection of the *kdr* allele in the *Culex pipiens* populations.

## Figures and Tables

**Table 1:** Mosquito sampling sites, south-eastern Romania, 2018–2019.

City/County	Site No.	Coordinates		Habitat
		N	E	
Bucharest	1	44°25′58.16″	26°05′01.22″	urban
	2	44°26′51.06″	26°02′42.90″	urban
	3	44°25′00.35″	26°06′48.61″	urban
	4	44°25′11.20″	26°08′24.51″	urban
	5	44°28′16.09″	26°03′01.46″	urban
	6	44°30′02.54″	26°04′11.40″	urban
	7	44°25′23.69″	26°01′48.63″	urban
	8	44°27′11.95″	26°05′02.98″	urban
	9	44°25′17.40″	26°04′55.95″	urban
Ilfov	10	44°23′48.51″	25°56′43.99″	rural
	11	44°22′45.52″	26°11′57.87″	rural
	12	44°20′23.37″	26°05′01.87″	rural
Giurgiu	13	44°05′50.31″	25°47′26.39″	rural
Prahova	14	45°11′16.53″	25°45′56.73″	rural
Tulcea	15	45°10′43.16″	28°49′00.67″	urban

**Table 2:** Distribution of acetylcholinesterase 1 (AChE1) F290V substitution in the mosquito samples analysed.

Location	*Culex pipiens pipiens*	*Culex pipiens molestus*	*pipiens* × *molestus* Hybrids
Bucharest and Ilfov county	98 SS	79 SS + 2 SR	15 SS
Giurgiu county	36 SS	9 SS	0
Tulcea county	22 SS	0	0
Prahova county	10 SS	0	0
Total number of specimens tested	166	90	15

S—allele encoding for phenylalanine (F) at codon 290 of AChE1; R—allele encoding for valine (V) at codon 290 of AChE1.

**Table 3:** Distribution of acetylcholinesterase 1 (AChE1) G119S substitution in the mosquito samples analysed.

Location	*Culex pipiens pipiens*	*Culex pipiens molestus*	*pipiens* × *molestus* Hybrids
Bucharest and Ilfov county	14 SS	13 SS + 2 SR	8 SS
Giurgiu county	3 SS	0	0
Tulcea county	2 SS	0	0
Prahova county	1 SS	0	0
Total number of specimens tested	20	15	8

S—allele encoding for glycine (G) at codon 119 of AChE1; R—allele encoding for serine (S) at codon 119 of AChE1.

**Table 4:** Distribution of voltage gated Na^+^ channel (VGSC) L1014F substitution in the mosquito samples analysed.

Location	*Culex pipiens* *pipiens*	*Culex pipiens molestus*	*pipiens* × *molestus* Hybrids
Bucharest and Ilfov county	12 SS + 41 SR + 45 RR	81 RR	2 SR +13 RR
Giurgiu county	14 SS + 16 SR +6 RR	9 RR	0
Tulcea county	17 SS +5 SR	0	0
Prahova county	10 SS	0	0
Total number of specimens tested	166	90	15

S—allele encoding for leucine (L) at codon 1014 of VGSC; R—allele encoding for phenylalanine (F) at codon 1014 of VGSC.

## Data Availability

Data can be provided on request from the corresponding author.
